# Protective Effects of Grapeseed Proanthocyanidins in Ulcerative Colitis: A Pilot Study Evaluating a Potential Therapeutic Strategy

**DOI:** 10.3390/jcm15020888

**Published:** 2026-01-21

**Authors:** Sonia Facchin, Elena Agostini, Elisa Laparra-Ruiz, Giuseppe Benvenuto, Giorgio Valle, Luisa Bertin, Edoardo Vincenzo Savarino

**Affiliations:** 1 Department of Surgery, Oncology and Gastroenterology (DISCOG), University of Padua, 35128 Padua, Italy; sonia.facchin@unipd.it (S.F.); luisa.bertin.1@phd.unipd.it (L.B.); 2 Gastroenterology Unit, Azienda Ospedale Università di Padova, 35128 Padova, Italy; elena.agostini_02@aopd.veneto.it; 3 BMR Genomics S.R.L., Via Redipuglia 22, 35131 Padova, Italy; elisa.laparra@bmr-genomics.it (E.L.-R.); giuseppe.benvenuto@bmr-genomics.it (G.B.); giorgio.valle@unipd.it (G.V.)

**Keywords:** inflammatory-bowel-disease, nutraceutics, ulcerative colitis, procyanidins, grape-seed-extracts

## Abstract

**Background/Objectives**: Recent research highlights *Vitis vinifera* seeds as a rich source of bioactive proanthocyanidins (PACs) with antioxidant and immunomodulatory effects. Poorly absorbed PACs are metabolized by gut microbiota into active phenolic metabolites. This pilot study in ulcerative colitis patients assessed grape seed extract effects on microbiota, zonulin-related permeability, and quality of life. **Methods**: This prospective pilot study, conducted at the University Hospital of Padua, evaluated the effects of an eight-week treatment with proanthocyanidins (ECOVITIS^®^) on gut microbiota, intestinal permeability (zonulin), and well-being in patients with ulcerative colitis in remission (IBDQ). Fecal and serum samples were collected at T0 and T1. Microbiota analysis was performed through 16S rRNA gene sequencing (QIIME2), zonulin was quantified using an ELISA kit for pre-haptoglobin gene2 (pre-HP2), and HP1/HP2 genotyping was conducted by quantitative PCR. Statistical analyses (Wilcoxon, ALDEx2, PERMANOVA) assessed microbial diversity and taxonomic changes between pre- and post-treatment samples. **Results**: Twenty-five ulcerative colitis patients completed the study. IBDQ scores significantly improved after treatment (mean Δ = +11.2, *p* < 0.001), especially in the 11 best IBDQ responders (Δ = +24.2, *p* < 0.001). Microbiota analysis showed increased *Lachnospiraceae* and *Sutterellaceae* in responders, while overall diversity remained unchanged. Zonulin levels were unaffected. **Conclusions:** PAC treatment improved quality of life in ulcerative colitis patients, as shown by increased IBDQ scores. Serum zonulin levels remained unchanged. Microbiota analysis revealed enrichment of *Lachnospiraceae* and *Sutterellaceae* families, suggesting beneficial modulation. Limitations include lack of metabolic assessment and a control group, and caution is needed in interpreting zonulin measurements.

## 1. Introduction

In recent years, both pharmaceutical research and economic strategies have increasingly focused on the investigation of secondary metabolites derived from plants as promising therapeutic agents for the prevention and treatment of non-communicable, lifestyle-related diseases. Among them, the seeds of *Vitis vinifera* L. have emerged as a particularly valuable source due to their complex matrix of bioactive compounds, which exhibit substantial pharmacological potential and could be leveraged for a variety of medical applications [1,2,3,4,5]. Indeed, its seeds are particularly rich in bioactive molecules [6], notably condensed flavan-3-ols, also known as proanthocyanidins (PACs), which have demonstrated antioxidant properties and are currently under investigation as potential therapeutic agents for the management of several chronic and severe diseases [7,8,9].

Proanthocyanidins are naturally occurring bioactive compounds found in the daily diet, especially in fruits and vegetables. Grape Seed Extracts (GSEs) are primarily composed of B-type PACs, which exhibit a variable degree of polymerization [10]. Due to their high molecular weight, these polymeric PACs are poorly absorbed in the small intestine. Consequently, the majority reach the colon, where they undergo extensive biotransformation by the gut microbiota, leading to the production of smaller, absorbable phenolic metabolites [11]. Several mechanistic studies have highlighted the direct immunomodulatory effects of PACs on immune cell signaling pathways, gut epithelial integrity, and even on the activity of isolated pathogenic organisms. It has been demonstrated that PACs can modulate host immune responses to bacterial, viral, and parasitic enteric infections, with gut microbiota modulation likely playing a mediating role in these effects [12].

A recent study investigated the effect of nutritional hormesis associated with the intake of antioxidant polyphenols [6]. The concept of hormesis is based on the biological phenomenon in which low doses of a potentially toxic or stress-inducing agent can have beneficial effects on the organism, whereas high doses are harmful. The authors proposed that the action of grape seed extract (GSE) polyphenols may be linked to a microbial catabolic effect, mediated by the production of a metabolite, 5-(3′,4′-dihydroxyphenyl)-γ-valerolactone (VL) [13]. Specifically, it seems that VL, after oxidation to its corresponding quinone, may activate the nuclear factor E2-related factor 2 (Nrf2) signaling pathway, stimulating antioxidant and immune defenses and helping to mitigate stress-induced inflammatory responses, such as those associated with TNF-α production.

It is well established that intestinal permeability is altered in IBD and is associated with “zonulin”, an endogenous protein that modulates tight junctions between epithelial cells. Overexpression of zonulin has been linked to increased intestinal barrier permeability (“leaky gut”), a condition frequently reported in patients with chronic inflammatory bowel diseases [14]. Zonulin has been proposed as the precursor of haptoglobin 2 (pre-HP2), a protein encoded by the *HP2* allele—one of the two main variants of the haptoglobin (*HP*) gene [15]. Haptoglobin (HP) is an acute-phase glycoprotein that binds free hemoglobin, thereby preventing oxidative damage [16,17]. Consequently, only individuals carrying at least one *HP2* allele (i.e., with *HP2-1* or *HP2-2* genotypes) have the genetic potential to express zonulin [15,18]. In the presence of factors that disrupt gut homeostasis, these individuals may exhibit elevated zonulin levels, which have been associated with impaired regulation of intestinal permeability [19].

In our prospective, single-center pilot study, we enrolled patients with ulcerative colitis (UC), a chronic inflammatory bowel disease (IBD) in which tumor necrosis factor-α (TNF-α) activity is well documented even during remission [20]. We administered a commercial formulation of proanthocyanidins and assessed its impact on the gut microbiota composition as well as on intestinal permeability, using the zonulin test, and patients’ well-being, using the Inflammatory Bowel Disease Questionnaire (IBDQ), before and after treatment.

## 2. Materials and Methods

This single-center, prospective, pilot study was conducted at Ospedale Università Padova, Gastroenterology Unit, between June 2024 and June 2025. We included 25 consecutive patients with UC, diagnosed according to ECCO current guidelines [21], who were clinically in remission at the time of the screening period. Since this is a pilot exploratory study, sample size calculation is not required. However, considering a recent investigation by our group where the administration of butyric acid was able to modify gut microbiota composition of approximately 90% of the patients, we considered it reasonable to hypothesize a similar change in gut microbiota composition after 2 months of Ecovitis. Moreover, using a Dirichlet multinomial model of 16S abundance data, we based power and sample size calculation on the open-source R statistical software package (R-4.5.2). Fixing α = 1% and a statistical power of 80%, a number of samples equal to 25 is necessary to detect significant abundant taxa and different alpha and beta diversity before and after treatment, assuming a response to treatment of 90% of subjects and a sequencing depth above 104 per sample. To estimate the parameters of the model, we used preliminary data available at our lab.

An oral formulation of Proanthocianidine >95% (ECOVITIS^®^, DISTILLERIE BONOLLO UMBERTO S.p.A, Mestrino, Italy) at a dose of two capsules/day (1 × 250 mg/cps carrying 150 mg of proanthocianidine) was administered, away from meals, for 8 weeks. At both time points, T0 (initial) and T1 (after 8 weeks), enrolled patients were requested to provide a serum sample and a fecal sample (e-Nat, Copan) and to complete the IBDQ at the corresponding study time points.

*Primary Outcome*: Given that the primary objective of this study was to assess changes in the gut microbiota following treatment with ECOVITIS, the primary outcome was defined as the 16S rRNA sequencing analysis. This method allowed for the evaluation of alpha and beta diversity, as well as the microbial composition of fecal samples at the genus level or higher.

*Secondary outcome*: To evaluate the clinical effects of PAC treatment, enrolled patients were asked to complete the Inflammatory Bowel Disease Questionnaire (IBDQ), a validated and widely used instrument to assess health-related quality of life in IBD patients [22]. The questionnaire explores several domains, including emotional well-being, intestinal and systemic symptoms, and social functioning, thereby providing a comprehensive measure of the impact of disease on patients’ daily lives. The use of the IBDQ in a clinical setting allows integration of conventional clinical and laboratory outcomes with a patient-centered parameter, which is particularly useful for monitoring treatment responses such as PAC administration. Patients were defined as responders if they improved their quality of life according to the IBDQ. In addition, the assessment of intestinal well-being (IBDQ) was found to be associated with changes in intestinal inflammation levels. Since it was not possible to evaluate variations in fecal calprotectin—given that only patients in remission were enrolled—serum zonulin measurement was employed in conjunction with the analysis of changes in the IBDQ score in order to combine a laboratory parameter and a clinical parameter related to UC.

The study was approved by the Regional Ethical Committee for Clinical Trials (No. 5908/AO/24 del 4 April 2024). Before participation, written informed consent was obtained from all eligible participants. The study was also registered at Clinicatrial.gov NCT06576700.

- **Quantification of serum zonulin by ELISA**

In order to quantify zonulin in serum, first an extensive search was carried out to identify an ELISA kit specifically designed for zonulin measurement. Kits that claimed to detect zonulin but whose technical specifications indicated the use of antibodies recognizing haptoglobin in any of its isoforms were excluded. Similarly, kits targeting zonulin-related proteins (ZRP) were also discarded.

Ultimately, the Elabscience Human Zonulin ELISA sandwich kit (E-EL-H5560) was selected, as the manufacturer confirmed that the capture antibody was specifically developed to recognize pre-HP2. Moreover, this kit has been used and referenced in 88 published articles. Zonulin levels were measured following the manufacturer’s instructions.

- **Genetic analysis of the *HP* gene**

The *HP* gene exists primarily in two allelic forms, *HP1* and *HP2*, which differ structurally due to an intragenic duplication of exons 3 and 4 in the *HP2* allele. As a result, *HP1* has five exons, while *HP2* contains seven, leading to the production of structurally distinct haptoglobin isoforms [23].

Genotyping of the *HP* gene was performed using a quantitative PCR approach based on the protocol developed by Soejima et al. (2008) [24], with some modifications introduced and previously validated by the BMR Genomics laboratory. This method enables reliable differentiation between *HP1* and *HP2* alleles by amplifying two specific regions of the *HP* gene: a region encompassing the duplication breakpoint of the *HP2* allele and the 5′ region of *HP* exon 1, used as an internal control [24].

Two separate master mixes were prepared using the Power SYBR^®^ Green PCR Master Mix protocol (Sigma-Aldrich-Italy and BMR genomics, Padova, Italy). For the *HP5* reaction mix: 5 µL of Power SYBR^®^ Green PCR Master Mix, 0.5 µL of 3 nM forward primer, 0.5 µL of 3 nM reverse primer, 2 µL of H_2_O, and 2 µL of 1 ng genomic DNA. For the *HP2* reaction mix: 5 µL of Power SYBR^®^ Green PCR Master Mix, 1 µL of 3 nM forward primer, 1 µL of 3 nM reverse primer, 1 µL of H_2_O, and 2 µL of 1 ng genomic DNA. The PCR cycle conditions were initial AmpliTaq Gold^®^ Enzyme (Thermo Fisher Scientific, Rome, Italy) activation at 95 °C for 10 m, 45 cycles of denaturation at 95 °C for 15 s, annealing at 59.5 °C for 30 s, and extension at 60 °C for 30 s. After amplification, genotypes were determined using the comparative Ct (ΔΔCt) method by quantifying the relative copy number of the 2-specific duplication, normalized to the promoter region as an internal reference [24,25].

- **Statistical Methods**

Categorical variables have been summarized with absolute frequencies and percentages. Continuous variables were described with appropriate position and dispersion indices.

The association between categorical variables was tested with the chi-square test or, if the criteria for its calculation were not met, with Fisher’s exact test. The association between continuous and dichotomous variables was tested with the t-test or the Mann–Whitney test. To test the distribution of the paired data, the Shapiro–Wilk test and the Wilcoxon signed-rank test were used.

The preprocessing analysis, the library preparation and sequencing, the bioinformatic processing, and the diversity analysis are described in Supplementary Information Briefly: Alpha diversity was assessed to evaluate within-sample microbial richness and evenness using Observed Features, Shannon entropy, the Simpson index, and the Chao1 richness estimator. Beta diversity was evaluated to characterize between-sample differences in community composition using Bray–Curtis dissimilarity and Jaccard distance metrics. Ordination was performed by Principal Coordinate Analysis (PCoA). Statistical differences in community structure between time points were tested using PERMANOVA, and homogeneity of dispersion was verified using betadisper and permutest functions implemented in the vegan R package.

## 3. Results

### 3.1. Patients’ Enrollment

Thirty consecutive patients were assessed for eligibility and enrolled on the day of routine endoscopy; two patients did not meet the inclusion criteria (as reported in Supplementary Information), having independently undergone antibiotic treatment within 10 days before enrollment. One patient declined to provide fecal samples and was therefore excluded from the study, while two others withdrew due to the need for antibiotic therapy for dental procedures.

A total of 25 patients completed the study and provided valid fecal samples, while only 24 patients provided valid blood samples. These numbers were considered sufficient for the statistical analysis of this pilot study. All enrolled patients met the inclusion criteria: they were adults (18–75 years) with the disease in remission as documented by surveillance colonoscopy, performed within one week before enrollment, without significant comorbidities, and able to comply with the study protocol.

Demographic characteristics of the entire study cohort (Overall column, N = 25), as well as of the subgroup of eleven patients who showed a clinically significant increase in IBDQ score at T1 (ΔIBDQ ≥ 17 as exploratory cut-off), are presented in Table 1.

### 3.2. Population and Zonulin Genotype Distribution

Of the 25 enrolled patients, genotyping and zonulin measurement were simultaneously performed in 24 patients. Statistical analyses were subsequently conducted on only 21 patients, excluding those with the HP1-1 genotype (Table 2). The distribution of paired differences deviated from normality (Shapiro–Wilk test, W = 0.913, *p* = 0.046). Two outliers were identified according to the 1.5 × IQR criterion (ID 11 and ID 16). After their removal, 19 data pairs remained, with T0-median = 82.27, IQR = 75.97, and T1-median = 99.86, IQR = 94.56. The distribution of paired differences was compatible with normality (Shapiro–Wilk test, W = 0.946, *p* = 0.340). A Wilcoxon signed-rank test showed no statistically significant difference between T0 and T1 (Z = 72.0, *p* = 0.557). The same result was obtained in the subset of eleven patients who were selected for a greater increase in IBDQ score (ΔIBDQ ≥ 17). All results are reported in Table 3.

### 3.3. IBDQ Analysis

**Figure 1:**
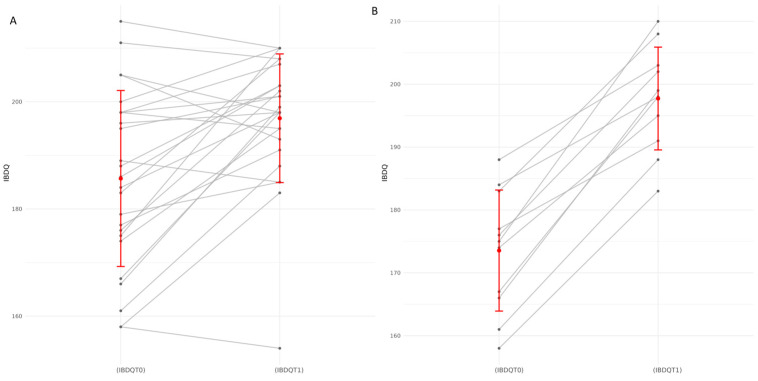
The figure shows the variations in the Inflammatory Bowel Disease Questionnaire (IBDQ) scores measured before (IBDQT0) and after (IBDQT1) treatment. Panel (**A**) reports the values for all patients, while Panel (**B**) reports the values for the subset of 11 selected patients.

The Inflammatory Bowel Disease Questionnaire (IBDQ) scores were compared before (T0) and after treatment (T1). Mean IBDQ scores increased from 185.7 ± 16.4 at baseline to 196.9 ± 12.0 following treatment (t = 4.04, *p* = 0.00048). The mean difference was +11.2 ± 13.9, corresponding to a large effect size (Cohen’s d = 0.81). These findings suggest that the treatment was associated with a significant enhancement in patients’ health-related quality of life (Figure 1).

### 3.4. Microbiota Analysis

The microbiota analysis conducted on the entire study population (N = 25), performed according to the procedures described in the Supplementary Information, did not yield statistically significant results in terms of alpha/beta diversity or discriminant taxa. To identify common characteristics among patients who reported a significant improvement in intestinal well-being—as assessed by the IBDQ—we investigated potential variations in gut microbiota composition. Thus, we selected eleven patients who exhibited a clinically meaningful increase in IBDQ (ΔIBDQ ≥ 17) scores following supplementation to undergo a taxonomic analysis at the family level.

Differential abundance analysis was performed using a paired study design, comparing samples collected at T0 and T1 for each subject. Two complementary inferential strategies were applied to ensure both interpretability and statistical robustness.

**Figure 2:**
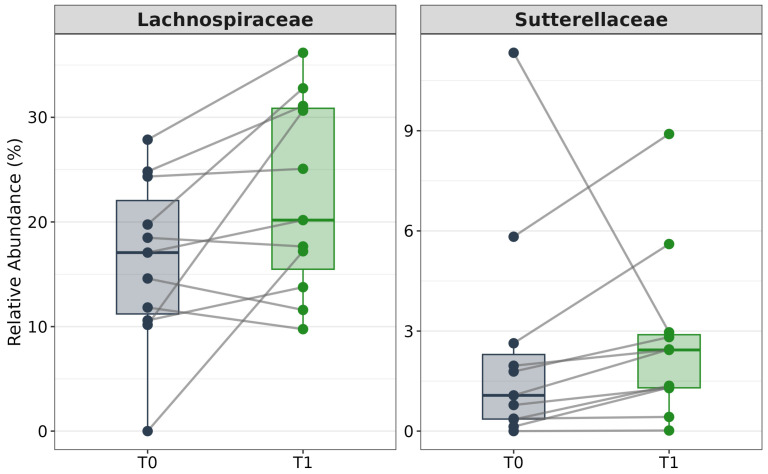
Paired slope charts display the individual trajectories of relative abundance for the *Lachnospiraceae* (**left** panel) and *Sutterellaceae* (**right** panel) families between baseline (T0, dark blue) and post-supplementation (T1, green). Gray lines connect paired samples from the same subject, visualizing the direction of change for each individual. Boxplots overlay the data points to represent the median and interquartile range of the group distribution. The analysis focuses on the subset of patients selected for exhibiting a robust clinical response. Both taxa show a consistent trend of enrichment following the intervention.

Using the paired Wilcoxon test to identify taxa showing significant changes between time points, a significant post-supplementation increase in the relative abundance of the *Lachnospiraceae* family was observed (Figure 2), suggesting an enrichment of metabolically beneficial species associated with gut health. Differential abundance analysis using ALDEx2 further identified the *Sutterellaceae* family as significantly altered (Table 4). ALDEx2 results were considered more statistically rigorous due to their compositional nature, while Wilcoxon-based results provided complementary, more intuitive metrics that facilitated biological interpretation.

## 4. Discussion

Grape seed extracts, which are rich in polyphenolic compounds, including oligomeric proanthocyanidins, have been extensively studied for their potential therapeutic effects across a range of health conditions. These include the prevention of cardiovascular diseases [3], management of chronic venous insufficiency (CVI), and the treatment of type 2 diabetes [5]. A growing body of clinical evidence supports the efficacy of GSEs in these contexts. Although monomeric and dimeric PACs undergo partial absorption along the gastrointestinal tract, higher-molecular-weight oligomeric and polymeric forms are essentially non-bioavailable [13].

However, these larger PACs interact actively with the colonic microbiota, promoting the generation of bioavailable metabolites that, through subsequent metabolic transformations, give rise to bioactive compounds capable of modulating various physiological processes. In this pilot study we aimed to evaluate the potential effects of GSEs in the treatment of chronic inflammatory bowel diseases, specifically in patients with UC. The study focused on assessing changes in the gut microbiota in relation to intestinal permeability and evaluating the impact on patients’ quality of life. Thus, we observed that treatment with PACs resulted in a significant improvement in patients’ perceived quality of life during clinical remission of UC, as demonstrated by the increase in IBDQ scores. Microbiota analyses were performed in this subgroup, since evaluations conducted in the entire study population did not reveal significant differences. Microbiota profiling showed a trend toward a modest, albeit limited, taxonomic improvement associated with the treatment; it showed a substantial increase in the *Lachnospiraceae* family, which includes several short-chain fatty acid (SCFA)–producing species and is generally associated with intestinal eubiosis. Differential abundance analysis further highlighted an enrichment of the *Sutterellaceae* family following PAC treatment. Although members of this family have historically been linked to pathological conditions [26], recent evidence has contributed to a reevaluation of their role within the gut microbiota. For instance, certain *Sutterella* species have been positively correlated with enhanced antibody responses following vaccination and have been shown to activate metabolic pathways associated with short-chain fatty acid (SCFA) and bile acid production [27]. Wang et al. [28] demonstrated that *Sutterella* may contribute to improved glycemic control and glucose homeostasis following surgical interventions. Moreover, a recent study by Dai et al. [29] reported that specific carotenoids promoted the expansion of *Sutterella,* suggesting its responsiveness to beneficial dietary compounds and its potential role in SCFA biosynthesis.

On the other hand, serum zonulin analyses did not provide evidence of an improvement in intestinal permeability following PAC treatment. Circulating zonulin levels are commonly measured as an indirect marker of intestinal permeability, particularly in clinical and research settings investigating gut barrier dysfunction. However, this does not imply that the patients experienced no improvement, as some inconsistencies were observed in zonulin levels relative to the patients’ HP genotypes. Specifically, individuals carrying the HP1 genotype were expected to show no measurable zonulin levels, since they are unable to produce it due to the absence of the HP2 allele.

We used a serum kit considered to be among the best commercially available and that has also been recently employed in publications in high-impact scientific papers [30,31,32]. Although the manufacturer reported that the ELISA kit used recognizes only the pre-HP2 and does not cross-react with other proteins, this was not reflected in the results. These discrepancies may reflect limitations in the assay’s specificity and raise concerns regarding the reliability of commercially available ELISA kits for zonulin quantification [33,34,35,36]. Furthermore, these findings call into question whether zonulin should be considered a reliable marker of intestinal permeability, as detecting or quantifying this protein in individuals with the HP1-1 genotype is, in principle, highly unlikely due to the absence of the HP2 allele. As previously reported by several authors [37], zonulin/pre-HP2 represents a true biological mediator of intestinal permeability; however, it is not a reliable biomarker in clinical practice, since its expression is genotype-dependent and its serum quantification may be methodologically fragile.

A key limitation of the present study is that we did not assess metabolic changes induced by dietary supplementation. However, to our knowledge, this is the first study to report microbial shifts associated with proanthocyanidin intake in conjunction with improvements in quality-of-life indices in patients with ulcerative colitis. An additional limitation of the present study concerns its design, as it was not conducted as a case–control study, which may reduce the overall robustness of the evidence obtained. In particular, the improvement observed in patients’ quality of life could be partly attributable to a placebo effect. However, this limitation appears to be mitigated by the fact that microbiota analyses performed in the same subgroup of patients actually documented an improvement in gut microbiota quality, suggesting the presence of biological changes consistent with the observed clinical benefits.

## 5. Conclusions

In this pilot study, PAC supplementation was associated with an improvement in quality of life in a subgroup of UC patients in clinical remission, as reflected by increased IBDQ scores. In the same subgroup, microbiota profiling revealed compositional changes, including an increased relative abundance of microbial families potentially beneficial to the intestinal ecosystem. Further adequately powered, controlled studies are warranted to confirm these observations and to clarify the clinical relevance of PAC supplementation in IBD, particularly in patients with active disease.

## Figures and Tables

**Table 1:** Characteristics of patients.

Demographic Variables	Overall, N = 25	Selection IBDQ, N = 11
**Sex (mean)**		
**F, (%)**	13, (52)	9, (81)
**M, (%)**	12, (48)	2, (19)
**Age** (geo-mean)	42.10	42.8
**Clinical variables (UC)**		
Disease remission (months) (*median*)	24	26.4
UC Montreal (E1/E2/E3)	2 (8%); 12 (48%); 11 (44%)	0 (0%); 7 (63%); 4 (36%)
**UC therapy (%)**		
5ASA	20 (80%)	8 (73%)
Biologics	12 (48%)	6 (55%)
Salazopyrine	2 (8%)	2 (19%)
Steroids	1 (4%)	1 (9%)
PPI	1 (4%)	0
Smokers	0%	0%
Previous abdominal surgery	0%	0%
Alcohol	2 (8%)	1 (9%)
**Physical activity (%)**		
None	7 (28%)	4 (36%)
Low	8 (32%)	4 (36%)
Medium	5 (20%)	3 (27%)
High	5 (20%)	0
**Comorbidity (%)**		
Hypertension	1 (4%)	1 (9%)
Hypercholesterolemia	4 (16%)	2 (19%)
Dysthyroidism	3 (12%)	2 (19%)
**Diet (%)**		
Omnivores	24 (96%)	10 (91%)
other	1 (4%)	1 (9%)

F = females; M = males; geo-mean = geometric mean; UC = ulcerative colitis; 5ASA = 5-aminosalicylic acid; PPI = proton pump inhibitor.

**Table 2:** Genotyping of the HP gene was successfully performed in all 25 individuals, revealing 12 individuals as HP2-2 (48%), 10 as HP2-1 (40%), and 3 as HP1-1 (12%). nr = blood sample not received.

No. Individual	Zonulin ng/mL T0	Zonulin ng/mL T1	Genotype
**1**	59.09	95.98	*HP2-2*
**2**	124.32	136.82	*HP2-1*
**3**	42.16	47.94	*HP2-2*
**4**	28.77	91.87	***HP1-1***
**6**	277.28	200.46	*HP2-2*
**7**	140.00	110.03	*HP2-1*
**8**	>1300	1270.04	***HP1-1***
**10**	208.48	257.14	*HP2-1*
**11**	283.11	407.67	*HP2-2*
**12**	97.62	147.10	*HP2-1*
**13**	27.54	27.12	*HP2-2*
**14**	63.64	59.58	*HP2-2*
**15**	>1300	>1300	*HP2-1*
**16**	204.01	409.34	*HP2-2*
**17**	82.27	66.43	*HP2-1*
**18**	83.53	nr	***HP1-1***
**19**	77.78	54.33	*HP2-2*
**20**	<1	11.72	*HP2-1*
**21**	337.00	345.17	*HP2-2*
**22**	117.13	46.26	*HP2-1*
**23**	53.30	99.86	*HP2-2*
**24**	16.50	19.41	*HP2-2*
**25**	72.09	144.29	*HP2-2*
**26**	106.58	103.33	*HP2-1*

**Table 3:** IBDQ score analysis.

	IBDQ T0 (Mean ± SD)	IBDQ T1 (Mean ± SD)	Mean Difference (± SD)	Paired *t*-Test (*p*-Value)
**IBDQ scores** **(N = 25**)	185.7 ± 16.4	196.9 ± 12.0	11.2 ± 13.9	<0.001
**IBDQ scores** **(N = 11)**	173.5 ± 9.6	197.7 ± 8.2	24.2 ± 7.5	<0.001

IBDQ: The Inflammatory Bowel Disease Questionnaire; SD: Standard Deviation.

**Table 4:** Microbiota analysis in responders to supplementation.

Family	Number of Patients	Median (T1-T0) *	*p*-Value	Delta Median (T1-T0) **	*p*-Value
***Lachnospiraceae***	11	+0.0318	**0.0322**	+0.0318	0.0843
***Sutterellaceae***	11	+0.0101	0.0537	+0.0101	**0.0263**

* Wilcoxon paired test; ** ALDEx^2^ paired test.

## Data Availability

The datasets used and/or analyzed during the current study are available from the corresponding author on reasonable request.

## Supplementary Materials

The following supporting information can be downloaded at: https://www.mdpi.com/article/10.3390/jcm15020888/s1, The data from the microbiota analysis, as well as the tables detailing the genetic characterization of zonulin, are included.

## Abbreviations

ELISA assays	Enzyme-Linked Immunosorbent Assay
GSEs	Grape Seed Extracts
HP	haptoglobin gene
IBD	Inflammatory Bowel Disease
IBDQ	Inflammatory Bowel Disease Questionnaire
NrF2	nuclear factor E2-related factor 2
PAC	Proanthocyanidins
SCFA	Short chain Fatty Acid
TNF-α	tumor necrosis factor-α
VL	valerolactoneDT Androgen Deprivation Therapy
