# Supplementary material for: Protective Effects of Grapeseed Proanthocyanidins in Ulcerative Colitis: A Pilot Study Evaluating a Potential Therapeutic Strategy
Source: J Clin Med. 2026 Jan 21;15(2):888. doi: 10.3390/jcm15020888 (PMC12841892; doi:10.3390/jcm15020888)
Supplement: Supplementary file 1 [file jcm-15-00888-s001.zip › jcm-4025612-supplementary.pdf]

Supplementary Materials

Protective Effects of Grapeseed Proanthocyanidins in Ulcerative Colitis: A Pilot Study Evaluating a Potential Therapeutic Strategy

Sonia Facchin, Elena Agostini, Elisa Laparra-Ruiz, Giuseppe Benvenuto, Giorgio Valle, Luisa Bertin and Edoardo Vincenzo Savarino

Pre-processing analysis

Library preparation and sequencing

For each sample, 5 μL of extracted DNA were used as template to amplify the V3–V4 region of the bacterial 16S rRNA gene following the protocol described by Takahashi et al., 2014, using modified primers compatible with Illumina sequencing adapters.
PCR products were checked on 1.5% agarose gel, purified using Thermolabile Exonuclease I (New England Biolabs), diluted 1:2, and reamplified in an indexing PCR with Nextera XT barcoded primers (Illumina, USA).
Following normalization with SequalPrep (Thermo Fisher Scientific), amplicons were pooled, purified with Agencourt XP beads (Beckman Coulter), and sequenced on an Illumina MiSeq platform using MiSeq Reagent Kit v3 (2×300 bp paired-end) chemistry.
All 22 initial samples met quality criteria for sequencing, and the twelve selected for this study generated approximately 1814516 raw paired-end reads, with a median frequency of 79754 reads for sample.

Bioinformatic processing

Sequence data were processed using QIIME2 v2024.10 (https://qiime2.org). Adapters and primers were removed using Cutadapt, and sequences were denoised using the DADA2 plugin (qiime dada2 denoise-paired) with the following parameters: trimming at 270 bp (forward) and 215 bp (reverse), quality filtering, merging of paired reads, dereplication, and chimera removal. This workflow produced 1223 Amplicon Sequence Variants (ASVs). Sequences shorter than 370 bp and those with relative abundance below 0.01% were filtered out to remove potential contaminants and spurious variants. After filtering, 519 ASVs associated with a total of 1350501 high-quality reads were retained in the final feature table.

Taxonomic classification of representative ASV sequences was performed using the SILVA 138.2 reference database (99% identity, Pro341F–Pro805R region) and the Naïve Bayes classifier implemented in QIIME2.

Diversity analysis

Alpha diversity was evaluated using Observed features, Shannon entropy, Simpson index, and Chao1 richness estimator.
Beta diversity was assessed through Bray–Curtis dissimilarity and Jaccard distance, followed by Principal Coordinate Analysis (PCoA).
Differences in community structure between groups and time points were tested using PERMANOVA (999 permutations) implemented in the vegan::adonis2()

function in R, while the homogeneity of group dispersion was verified using betadisper and permutest functions.

All integrative statistical analyses were conducted in R (v4.4.0) using the packages phyloseq, vegan, ggplot2, ANCOMBC, and ALDEx2. After importing the feature table and metadata from QIIME2, the phyloseq object was constructed and taxonomic features were collapsed at the Family level to obtain aggregated abundance profiles suitable for comparative testing.

Before microbiota-level comparisons, individual changes in IBDQ (ΔIBDQ) were calculated for each subject, and the upper tertile cutoff (ΔIBDQ ≥ 17) was selected to identify individuals showing the strongest clinical improvement. This classification allowed subsequent analyses to account for the magnitude of response while maintaining a consistent paired design.

Differential abundance analysis was performed using a paired study design, comparing samples collected at T0 and T1 (column Tempo) for each subject. Two complementary inferential strategies were applied to ensure both interpretability and statistical robustness. First, paired Wilcoxon signed-rank tests were performed on raw relative abundances to identify taxa showing significant changes between time points. For each family, the median difference in relative abundance (T1–T0) was computed, and both raw and false discovery rate–adjusted p-values (Benjamini–Hochberg correction) were reported. Relative abundance values were retained for interpretive purposes and visualization, providing an intuitive representation of the direction and magnitude of change across taxa.

In parallel, the same comparisons were carried out within a compositional data framework using ALDEx2, which models sequencing count variability through Monte Carlo sampling of the centered log-ratio (CLR) transformation. ALDEx2 results were considered more statistically rigorous due to their compositional nature, while Wilcoxon-based results provided complementary, more intuitive metrics that facilitated biological interpretation.

All statistical outcomes, including p-values, FDR-corrected q-values, and median changes in relative abundance, were summarized at the family level and used to support biological interpretation of the microbiota shifts observed between baseline and post-treatment samples.

Inclusion criteria:

- Diagnosis of UC confirmed by clinical endoscopic and histopathological evidence evidence. Disease in remission phase confirmed by clinical (IBDQ 32 ≥ 170),, endoscopic (Mayo score = 0) evidence.
- Age between 18 and 75 years old
- Ability of subject to participate fully in all aspects of this clinical trial.

Exclusion criteria:

- Patients that have active UC, determined by clinical, endoscopic and histopathological evidences
- Known diagnosis of CD, indeterminate colitis, ischemic colitis, radiation colitis, diverticular disease associated with colitis or microscopic colitis
- Positive stool culture for active *C. difficile*
- Pregnant women
- Patients under antibiotic and/or probiotic treatment within 10 days prior to Visit 1.
